# The Effects of Socioeconomic and Environmental Factors on the Incidence of Dengue Fever in the Pearl River Delta, China, 2013

**DOI:** 10.1371/journal.pntd.0004159

**Published:** 2015-10-27

**Authors:** Xiaopeng Qi, Yong Wang, Yue Li, Yujie Meng, Qianqian Chen, Jiaqi Ma, George F. Gao

**Affiliations:** 1 National Center for Public Health Surveillance and Information Services, Chinese Center for Disease Control and Prevention, Beijing, China; 2 State Key Laboratory of Resources and Environmental Information System, Institute of Geographical Sciences and Natural Resources Research, Chinese Academy of Sciences, Beijing, China; 3 Office of the Director, Chinese Center for Disease Control and Prevention, Beijing, China; Australian National University, AUSTRALIA

## Abstract

**Background:**

An outbreak of dengue fever (DF) occurred in Guangdong Province, China in 2013 with the highest number of cases observed within the preceding ten years. DF cases were clustered in the Pearl River Delta economic zone (PRD) in Guangdong Province, which accounted for 99.6% of all cases in Guangdong province in 2013. The main vector in PRD was *Aedes albopictus*. We investigated the socioeconomic and environmental factors at the township level and explored how the independent variables jointly affect the DF epidemic in the PRD.

**Methodology/Principal Findings:**

Six factors associated with the incidence of DF were identified in this project, representing the urbanization, poverty, accessibility and vegetation, and were considered to be core contributors to the occurrence of DF from the perspective of the social economy and the environment. Analyses were performed with Generalized Additive Models (GAM) to fit parametric and non-parametric functions to the relationships between the response and predictors. We used a spline-smooth technique and plotted the predicted against the observed co-variable value. The distribution of DF cases was over-dispersed and fit the negative binomial function better. The effects of all six socioeconomic and environmental variables were found to be significant at the 0.001 level and the model explained 45.1% of the deviance by DF incidence. There was a higher risk of DF infection among people living at the prefectural boundary or in the urban areas than among those living in other areas in the PRD. The relative risk of living at the prefectural boundary was higher than that of living in the urban areas. The associations between the DF cases and population density, GDP per capita, road density, and NDVI were nonlinear. In general, higher “road density” or lower “GDP per capita” were considered to be consistent risk factors. Moreover, higher or lower values of “population density” and “NDVI” could result in an increase in DF cases.

**Conclusion:**

In this study, we presented an effect analysis of socioeconomic and environmental factors on DF occurrence at the smallest administrative unit (township level) for the first time in China. GAM was used to effectively detect the nonlinear impact of the predictors on the outcome. The results showed that the relative importance of different risk factors may vary across the PRD. This work improves our understanding of the differences and effects of socioeconomic and environmental factors on DF and supports effectively targeted prevention and control measures.

## Author Summary

Dengue fever is an infectious disease transmitted by mosquitoes. It is a major public health problem in tropical and subtropical regions around the world. Dengue fever is of great interest in the Pearl River Delta economic zone (PRD) of Guangdong province, China because the outbreak in 2013 was the largest in the previous 10 years. Due to the low degree of diversity in the climatic conditions in the PRD, socioeconomic and environmental factors may be the major contributing factors. The objective of this paper was to perform an assessment and detect the socioeconomic and environmental impact on cases at the smallest administrative unit (the township level). Six factors were identified in this work, representing urbanization, poverty, accessibility and vegetation. The effects of all these factors were found to be significant. The results showed that the relative importance of different risk factors may vary across the PRD. The higher risk areas and vulnerable populations identified in this paper will provide guidance for public health practitioners to create targeted, strategic plans and implement effective public health prevention and control measures.

## Introduction

Dengue fever (DF) is an infectious epidemic disease that is principally transmitted by the vector *Aedes albopictus* and *Aedes aegypti*.[1] Dengue virus infection in humans is often asymptomatic but can lead to different clinical manifestations.[2] In the past 50 years, the incidence of DF has increased 30-fold with increasing geographic expansion into new countries.[3] In tropical and subtropical regions around the world, the disease causes great concern. The rapid spread of DF has been attributed to a combination of urbanization, globalization and a lack of effective mosquito control.[4] Presently, there are no effective vaccines or specific therapies to stop the rapid worldwide spread of DF.[5] The heterogeneity of DF incidence in time and space is related to many risk factors, such as geography, environment and socioeconomic status. Some studies have mapped the spatial and temporal clustering patterns of DF cases.[6, 7] Other studies have analyzed the association between these patterns and relevant factors (e.g., precipitation, humidity, and temperature).[8, 9] Moreover, some studies have emphasized the relationship between socioeconomic factors and DF.[10]

In mainland China, DF cases have been reported every year since 1997, particularly in the Guangdong province.[11, 12] Between 2001 and 2010, a cluster of DF cases in the Guangdong province occurred in the Pearl River Delta economic zone (PRD).[13, 14] The main vector in PRD was *Aedes albopictus* based on the routine sentinel vector surveillance. Although these studies have helped us understand the mechanism of DF epidemics on a large scale in mainland China, the main socioeconomic and environmental factors in the PRD area (in addition to climate factors on a smaller scale) have not been identified. The relative importance of different risk factors may vary across countries and regions. Due to the low level of diversity in the climatic conditions in the PRD of China, socioeconomic and environmental factors may largely contribute to the spatial heterogeneity of the DF incidence in this area.

There was a great increase in the incidence of DF in the Guangdong Province in 2013. The number of DF cases that year was the highest yet of the previous 10 years and exceeded the total number of cases over the previous 6 years. The DF cases in PRD accounted for 99.6% of the total number of DF cases in the Guangdong Province in 2013. The objective of this paper was to perform an assessment of the socioeconomic and environmental impacts on DF cases between different neighborhoods in the PRD of China in 2013 at the smallest administrative unit—the township level (the hierarchal levels of Chinese administrative region are National, Province, Prefecture, County and Town). This is the first study to identify the relationship between these factors and DF cases on a small scale in mainland China.

## Methods

### Study area

**Fig 1:**
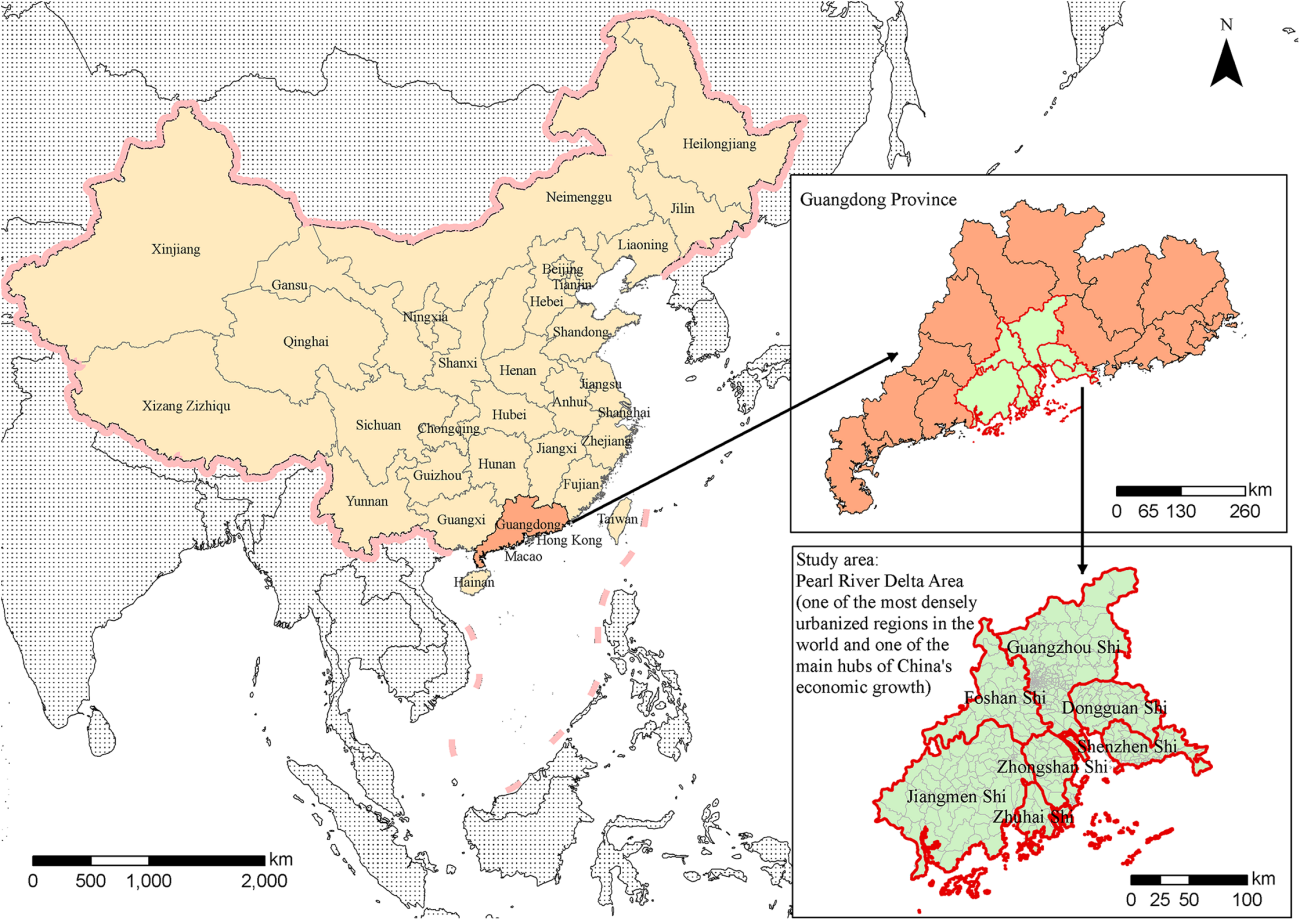
Study area.

The PRD in the Guangdong province is located at the Pearl River estuary, where the river enters the South China Sea. The province comprises seven cities (Guangzhou, Shenzhen, Dongguan, Foshan, Zhongshan, Zhuhai and Jiangmen) and parts of Huizhou and Zhaoqing (see Fig 1). It has been the most economically dynamic region of the Chinese mainland since the launch of China's reform program in 1979. It is one of the most densely urbanized regions in the world and one of the main hubs of China's economic growth. In our study, the PRD included only the first seven cities, with 402 streets and towns (excluding four island-towns), where the DF cases frequently occurred.

### DF data

Data on the DF cases were obtained from the China Information System for Disease Control and Prevention, which was developed by the Chinese Center for Disease Control and Prevention (China CDC) in 2004. The targeted DF cases in our study included clinically diagnosed (based on clinical manifestations and epidemiologic exposure history) or laboratory-confirmed cases (“clinically diagnosed cases presenting with any of the following lab test results relating to DF: a 4-fold increase in specific IgG antibody titer, positive on a PCR test or viral isolation and identification test”).[13] The date of DF onset was in 2013 and the permanent residential addresses of the diagnosed individuals were within the PRD.

### Socioeconomic and environmental data

#### Urbanization (urban environment)

Three factors were extracted to describe different aspects of urbanization, including “street/town at the prefectural boundary or not” (abbreviation “boundary”), “urban & rural” and “population density” in the PRD. Streets and towns are at the same level in China. Although the areas are divided into urban and rural areas at the level of community/village according to the classification criteria of the National Bureau of Statistics of China, the village data were not accessible in this study. Generally speaking, “streets” are usually located in urban areas and “towns” are usually located in the rural areas in China, which allows an approximate differentiation of the urban and rural situations in the PRD. “Population density” was calculated using the number of people living permanently in the street/town in 2013 divided by the area (km^2^).

#### Poverty

Data on the gross domestic product (GDP) per capita in 2010 at the township level in the PRD were from the National Bureau of Statistics of China. The average GDP in the PRD is 77,000 CNY (12,000 USD).

#### Accessibility

In our paper, road density was used to indirectly reflect the accessibility of the area in the PRD. Roads are classified based on their administrative and utility roles. In China, a functional approach has been adopted with the hierarchy of roads based on size, use for mobility and accessibility parameters. China categorizes roads into five classes including highway and Class 1–4 with the descending volume. Class 4 is a local road at the township and village level, which has little effect on regional movement. The highway and Class 1–3 roads in 2011 were adopted in our study to calculate the road density.

#### Environment

The Normalized Difference Vegetation Index (NDVI) is a simple graphical indicator (measured by satellite remote sensors) that can be used to assess whether the observed target area contains live green vegetation. The NDVI value can range from -1 to +1, with high values corresponding to a dense and active vegetation cover. The mean NDVI in each street/town in 2011 was used in this study. These data were obtained from the Institute of Geographic Sciences and Natural Resources Research, CAS, in China.

**Fig 2:**
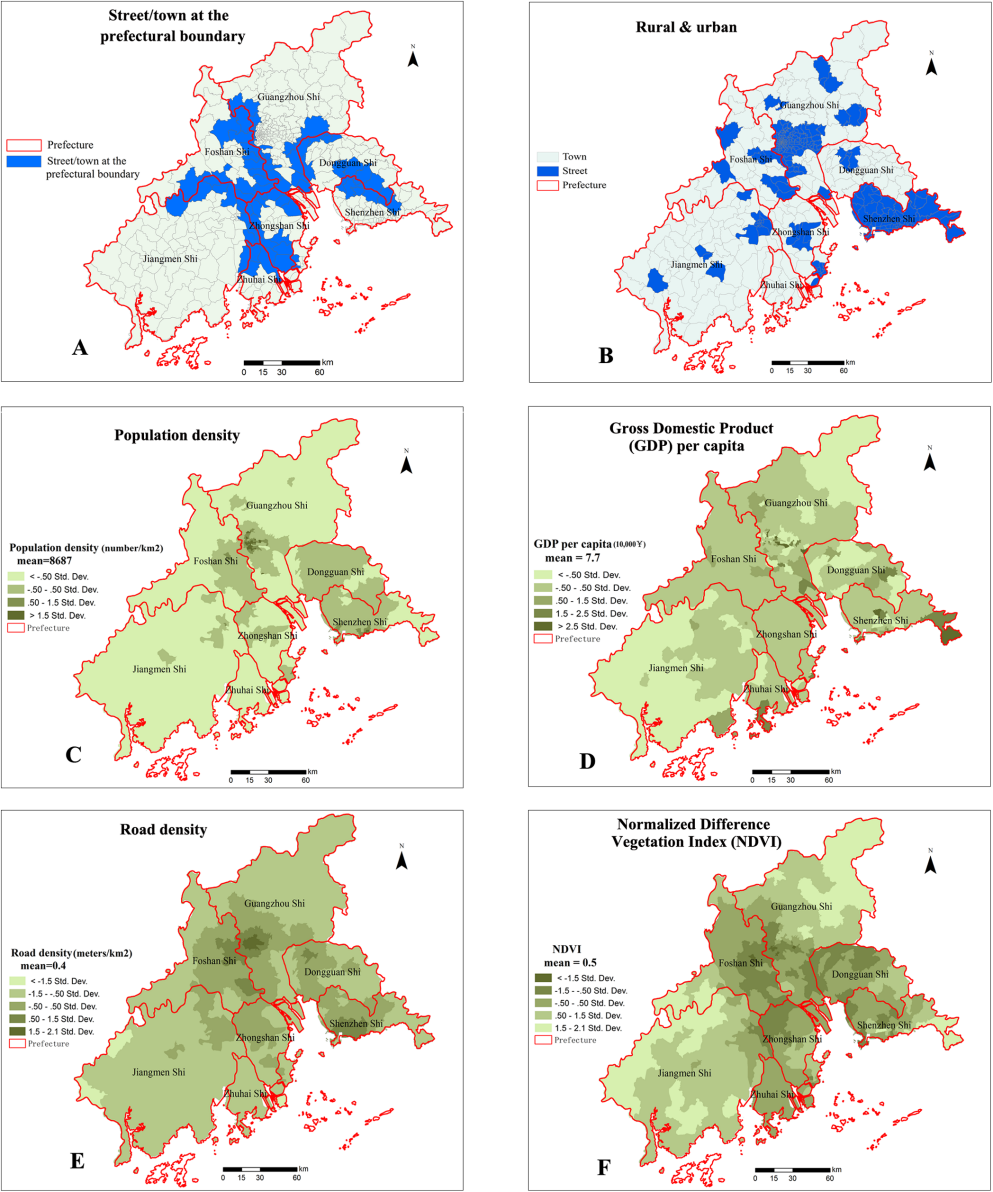
Spatial distribution of socioeconomic and environmental data at the township level in the PRD, China. A: Street/town at the prefectural boundary; B: Rural and urban; C: Population density; D: Gross Domestic Product (GDP) per capita; E: Road density; F: Normalized Difference Vegetation Index (NDVI).

The spatial distribution of the six exploratory variables is mapped in Fig 2. Fig 2A shows the distribution of the street/town at the prefectural boundary, Fig 2B shows the distribution of streets and towns, Fig 2C shows the population density, Fig 2D shows the distribution of GDP per capita, Fig 2E shows the road density, and Fig 2F shows the NDVI distribution.

### Statistical analysis

Analyses were performed with Generalized Additive Models (GAM) to fit parametric and non-parametric functions to the relationships between the response and predictors. GAM is a statistical model that extends the generalized linear models to include nonparametric smoothing terms.[15] To evaluate the possible non-linearity of the socioeconomic and environmental effect on the dengue cases, we used a spline-smooth technique and plotted the predicted against the observed co-variable values. We specified the expected number of DF cases as follows:

$$\log\left(case\right)=\beta_{0}+\beta_{1}(boundary)+\beta_{2}(urban\&rural)+s(\mathrm{pop}\_\mathrm{density})+s(\mathrm{GDP}\_\mathrm{per}\_\mathrm{capita})+s(\mathrm{road}\_\mathrm{density})+s(\mathrm{NDVI})$$

where s() is the spline smooth, non-parametric function. The “street/town at the prefectural boundary” and “urban & rural” variables were binary, which fit with the parameter function. “Population density,” “GDP per capita,” “road density” and “NDVI” were nonlinear and fit with the spline smooth technique using specific degrees of freedom (df) for each smoothing, depending on visual inspections of the estimated curves for a range of choices of smoothing (if there are more df, do not add an obvious structure to the curves, and smaller df change the shape of the curve, and the ‘optimal’ df have been reached).[16] All statistical analyses were performed using the statistical software R 3.0.3,[17], with the mgcv library.[18]

## Results

**Fig 3:**
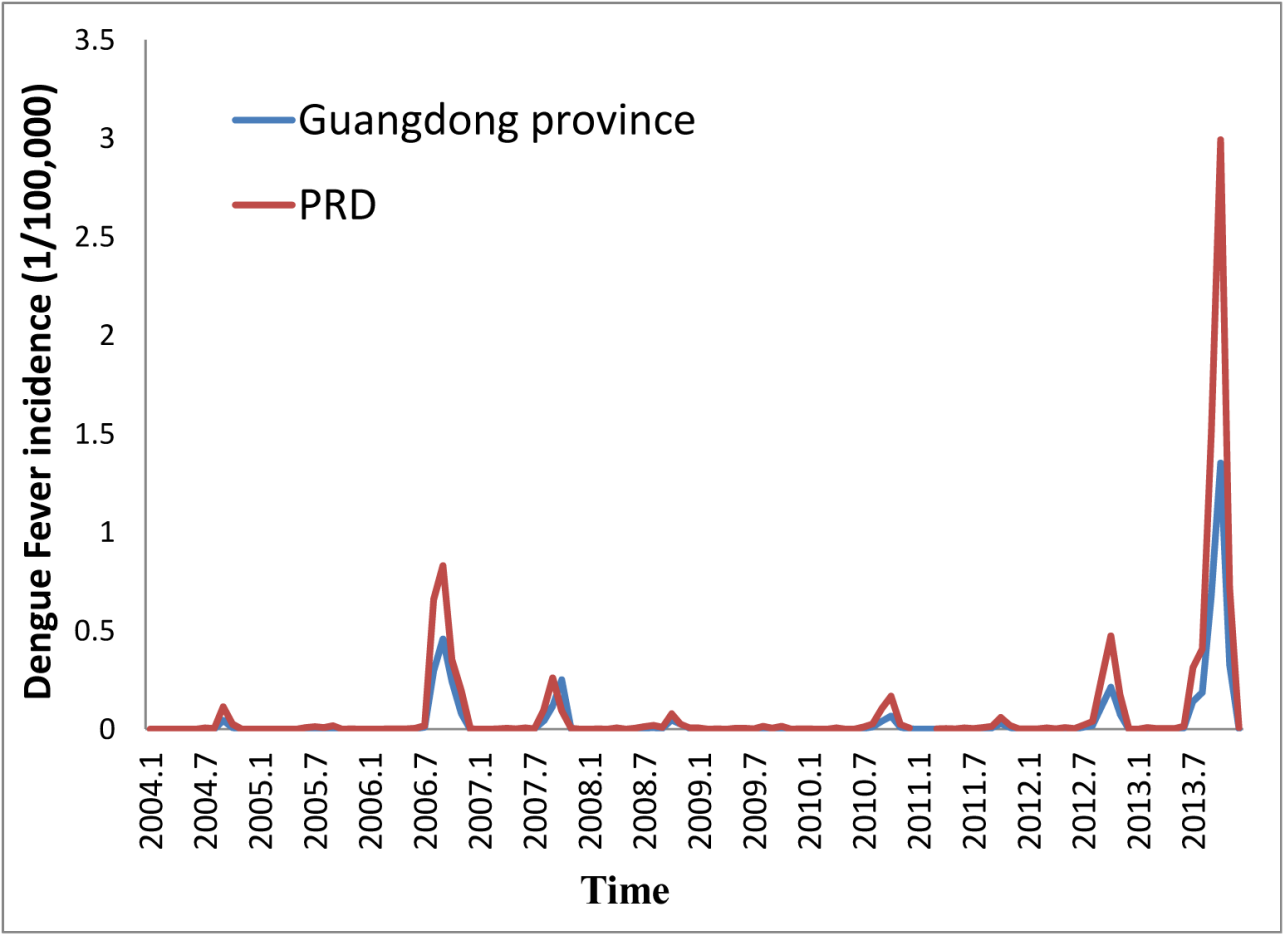
The monthly DENGUE FEVER incidence rate (1/100,000) in the PRD and the Guangdong Province, China, 2004–2013.

DF cases in Guangdong province principally cluster in the PRD. Fig 3 describes the monthly DF incidence rate in the PRD and the Guangdong Province, China from 2004 to 2013. The epidemic peaks were similar in the Guangdong province and the PRD. The highest monthly incidence was observed in October 2013, at 3 per 100,000 in the PRD, which was 2.2-fold higher than the incidence in the Guangdong province. The incidence of DF in 2013 in the PRD was approximately 6 per 100,000, which was much higher than the national incidence in 2013 (0.34 per 100,000).

**Fig 4:**
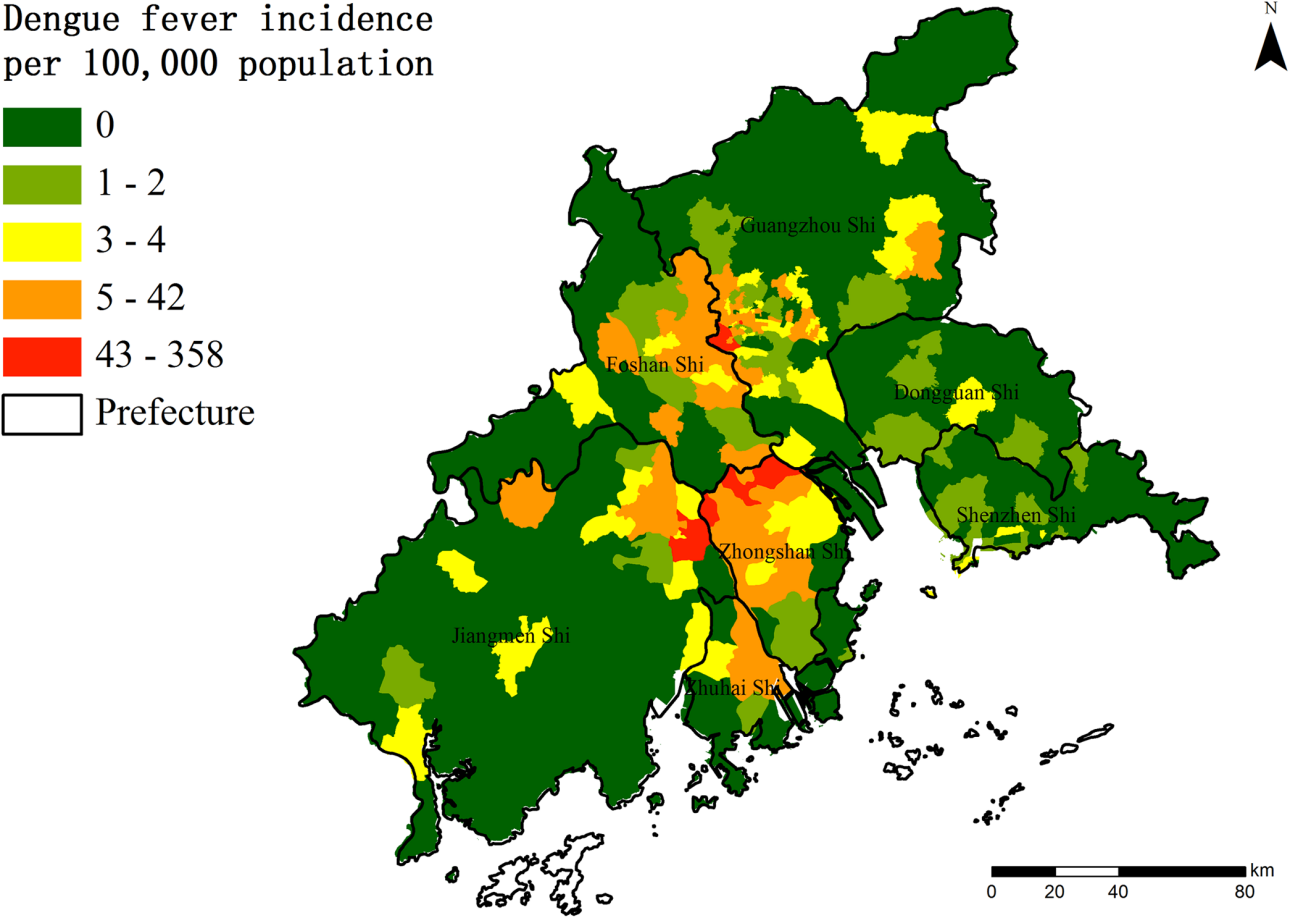
The spatial distribution of DENGUE FEVER incidence at the township level in the PRD, China, 2013.

A total 2889 DF cases were clinically diagnosed or laboratory confirmed in the PRD in 2013. Fig 4 shows the geographic distribution of targeted DF incidence at the township level in the PRD. The color strip from green to red indicates the increasing levels of DF incidence. The cases were highly clustered in the middle of the PRD. No DF cases occurred in nearly half of the streets/towns. According to the criteria for assessing the goodness of fit for the dengue cases (Pearsonχ^2^ /df close to 1), the distribution of dengue cases was over-dispersed (Poisson Pearsonχ^2^ /df = 108.67) and fit the negative binomial function (Pearsonχ^2^ /df = 2.60) better. Thus, the log link function for a negative binomial distribution response was selected in our study. The method for negative binomial response data is used to decide which terms to include in the model. The AIC (Akaike Information Criterion) /UBRE(Un-Biased Risk Estimator) scores for the models were compared with and without the term. The value yielding the lowest AIC/UBRE was selected in the study. Finally, all six factors were entered into the model. We specified an offset for the predictor and modeled the counts as negative binomial variables with the logarithm of the population as the offset variable.

Tables 1–3 show the estimates of the negative binomial GAM for DF cases per street/town in the PRD and the diagnosis information of the model. The effects of all socioeconomic and environmental variables were found to be significant at the 0.001 level. This specification of the model explained 45.1% of the deviance of the DF incidence.

**Table 1:** Linear terms effect of the negative binomial GAM model.

	Estimate	Std. Error	z value	Pr (>|z|)
(Intercept)	-11.1037	0.1496	-74.229	**< 2e-16**
Boundary	0.8288	0.1651	5.019	**5.2e-07**
Urban & rural	0.6905	0.1832	3.769	**0.000164**

Notes: Values in bold font were significant at the 0.001 level

**Table 2:** Approximate significance of smooth terms of the negative binomial GAM model.

	edf	Ref.df	Chi.sq	p-value
s(pop_density)	1.981	2.000	60.70	**6.62e-14**
s(GDP_per_capita)	3.796	3.974	18.82	**0.000844**
s(road_density)	2.969	2.999	116.25	**< 2e-16**
s(NDVI)	2.991	3.000	166.17	**< 2e-16**

Note: Values in bold font were significant at the 0.001 level

edf >1 indicate nonlinear relationships

**Table 3:** The negative binomial GAM model statistics diagnosis.

N:	402
Deviance explained:	0.451
UBRE score (sp.criterion):	1.0346
AIC	1901.008
Scale estimate:	1
family:	Negative Binomial(1.001)
link function:	log

The DF incidence showed a significant positive association with “boundary” and “urban & rural,” indicating that there is a higher risk of DF for people living at the prefectural boundary or in urban areas than for those living in other places in the PRD. The associations between DF cases and population density, GDP per capita, road density, and NDVI were nonlinear, according to the edf value (effective degrees of freedom of the smooth function terms).

**Fig 5:**
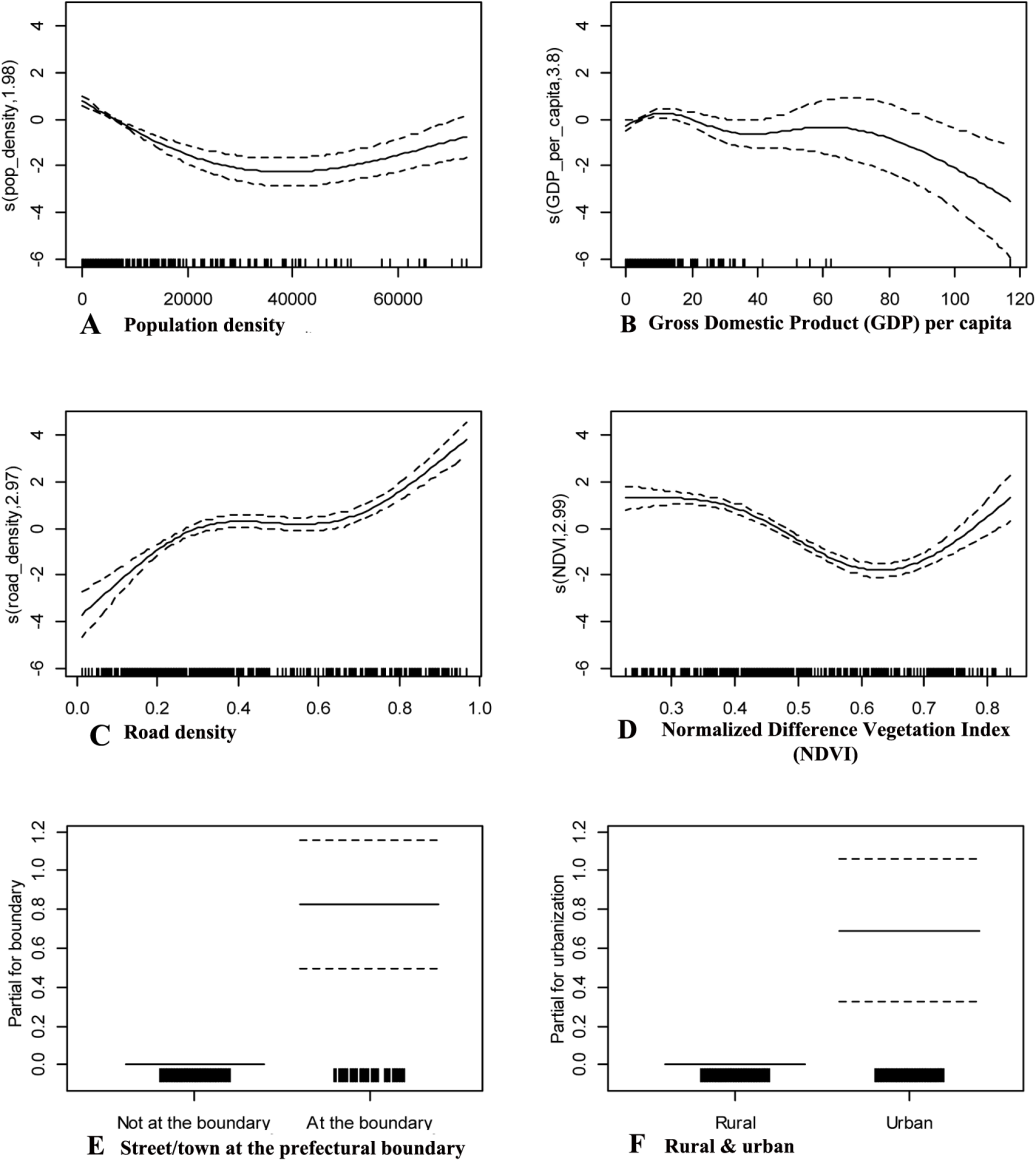
Partial contributions of six exploratory variables with confidence bands. A: Population density; B: Gross Domestic Product (GDP) per capita; C: Road density; D: Normalized Difference Vegetation Index (NDVI); E: Street/town at the prefectural boundary; F: Rural and urban). Smoothed functions (solid line) are bounded by 95% point wise standard errors (dashed lines for curves).

The partial contributions of six covariates to the conditional probability of DF cases with confidence bands are shown in Fig 5. Fig 5A shows the parabolic curvature of the population density and reveals that areas with 30,000–40,000 people per square kilometer are under lower risk than areas with higher or lower population densities. Fig 5B shows small fluctuations around the zero response of DF to GDP per capita below 400,000 CNY (65,000 USD), a modest, stable response above this threshold, followed by a rapid decline response when GDP per capita rises above 600,000 CNY (97,000 USD). Thus, the higher GDP per capita, the lower the risk of DF occurrence. Fig 5C depicts a positive relationship between the risk of DF and road density with little further benefit of road density between 0.4 and 0.7. This result indicates that high area accessibility can increase the risk of DF. The risk of DF declines gradually with rising NDVI (Fig 5D), showing a curve trough at approximately 0.65, after which the response increases. Fig 5E and 5F show that the relative risk of living at the prefectural boundary is higher than that of living in urban areas.

## Discussion

In this study we presented an effect analysis of socioeconomic and environmental variables on DF occurrence in a small scale of the PRD of China, 2013. Furthermore, we used the GAM statistical method that integrates parametric and non-parametric terms. GAM is specifically designed to analyze data when the impact of the predictors on the outcome is nonlinear. The results of this study demonstrate the power of GAM to reveal meaningful curvatures in exploratory analyses.

Generally, temperature and precipitation were regarded to be the main contributors to the occurrence of DF.[5, 19, 20], but they were not included in the model because we compared the temperature and precipitation values within the PRD in 2013 and found little differences in space. We focused on the DF cases in 2013 because there was a large outbreak in the PRD that year. The local, quick spread of DF in 2013 was associated more with socioeconomic and local environmental diversity instead of climate factors, which might increase the accuracy of the model.

Risk factors were selected based on the following rationale. Urbanization: DF transmission has been reported in both rural and urban areas. However, urban environments are characterized by many factors, such as a higher population, poor hygiene, poor housing conditions and less environmental management.[5, 21, 22] Rapid urbanization with large populations living in peri-urban slums provides attractive features for the *Aedes* mosquito and promotes DF transmission.[23]. Since we did not have access to a single indicator to reflect urbanization, we used “street/town at the prefectural boundary”, “street and town” and “population density” to indicate urbanization in China from different perspectives. Poverty: Several studies have linked poverty or relative poverty to DF. Poorer areas are characterized by factors that may favor higher DF transmission.[24] The GDP per capita is often considered to be an indicator of a country's standard of living. Accessibility: DF virus introduction and reintroduction are usually caused by individual human movements.[25] The movement of infected humans results in different patterns of spatial distribution. Therefore, human movement is one of the key elements that promotes the spread of DF, particularly in areas with more broad highways and interconnected roads. Environment: A close association between the local climate, vegetation and breeding mosquitos is usually identified. Indeed, vegetation can be the index indicator that “provides resting or feeding sites for mosquitoes or can serve as a proxy for the presence of breeding sites.”[26].

Many socio-environmental indicators related to DF occurrence occur on a small scale, such as water storage, the frequency of garbage collection, and the type of sewage disposal, which suggests precarious housing conditions and poor access to public services.[27] Six macroscopic socioeconomic and environmental factors were adopted for our model and found to be statistically significant. Although the vector’s proliferation is not directly related to these six factors, they indirectly reflect the urbanization, poverty, accessibility and environment and need to be valued by public health practitioners. “Urban areas,” “higher road density” and “lower GDP per capita” are considered to be the consistent risk factors in the PRD in the results and are consistent with the findings in previous studies.[5, 28, 29] “Population density” and “NDVI” are relatively complicated in that higher or lower values are both dangerous. This finding verifies that the relative importance of different risk factors may vary across the PRD. For example, high DF incidence in areas with lower population density may be caused by the higher NDVI, which provides a microenvironment for the proliferation of mosquitoes. “Street/town at the prefectural boundary” in PRD was identified in this paper as one of the risk factors. In China, “Street/town on the prefectural boundary” can roughly be regarded to be peri-urban with less management from both prefectures because most of the streets/towns on the prefectural boundary are far away from the prefectural centers. These peri-urban areas can easily cause poor hygiene and indirectly promote the vector cluster. Usually, poor hygiene and crowded populations can contribute together to increase the number of DF cases. This finding was proved in our model.

Therefore, the identification of the socioeconomic and environmental factors and the partial trend shown in the model in this paper provide meaningful clues for epidemic assessment and local interventions to counteract risk factors. For instance, cases are associated with low population density and vegetated areas would suggest that environmental hygiene measures would be ineffective and targeted Aedes albopictus eradication in vegetated areas will work. High population density areas especially at the prefectural boundary and urban areas may respond to "clean up" campaigns in advance. Focusing more resources on particularly vulnerable areas of the city can aid in strengthening people’s awareness of defense. It can also facilitate timely response strategies during a DF outbreak by pointing to areas that are more vulnerable, with the objective of minimizing the epidemic speed of the virus.

However, generalization of the results and causal inference are difficult because this was an ecological study and the study is subject to ecological bias.[30] The differences in vector density, preventive strategies, educational scope, and interventions applied in each epidemic area were not considered. These factors may be diverse, resulting in the heterogeneous distribution of DF cases. Thus, the study may contain errors that are inherent in reported case data and in the use of these data sources for epidemiological studies. We did periodically report data quality investigations and found some differences in the underreporting rate between each area. Because the investigational areas are representative of only the provincial level and cannot be used to adjust the cases in our study area, future targeted data quality investigations in the PRD should be conducted.

Although the DF cases in this study occurred in 2013, some of the data on the influential factors were collected in different years, including the GDP per capita in 2010, road density in 2011 and the NDVI in 2011. However, the lack of data in the corresponding year will have little impact on the results because the proportion of these three indicators in the PRD may change little within 2–3 years. Concurvity (the nonparametric analogue of multicollinearity) may be present in the data, leading to significance tests with an inflated type 1 error.[31] The values of the estimated indices of concurvity for four nonparametric terms in this study ranged from 0.15 to 0.73.

In conclusion, a complex relationship between socioeconomic environmental factors and DF occurrence was found in the PRD of China. Additive models offer flexible modeling tools for regression problems. Understanding the role of the socioeconomic and environmental factors in DF occurrence has important implications for the planning and implementation of effective public health prevention and control measures.[32] Targeted strategic planning could focus on vulnerable populations and the higher risk areas, which are consistently affected each year in the future.
